# Protocols for Dual Tracer PET/SPECT Preclinical Imaging

**DOI:** 10.3389/fphy.2020.00126

**Published:** 2020-05-08

**Authors:** Julia E. Blower, Jayanta K. Bordoloi, Alex Rigby, Matthew Farleigh, Jana Kim, Hugh O’Brien, Jonathan Jackson, Constantinos Poyiatzis, James Bezer, Kavitha Sunassee, Philip J. Blower, Lefteris Livieratos

**Affiliations:** School of Biomedical Engineering and Imaging Sciences, King’s College London, St Thomas’ Hospital, London, United Kingdom

**Keywords:** SPECT, PET, radionuclide, phantom, multi-modality, dual-radionuclide, dead-time, scatter

## Abstract

**Background:**

Multi-tracer PET/SPECT imaging enables different modality tracers to be present simultaneously, allowing multiple physiological processes to be imaged in the same subject, within a short time-frame. Fluorine-18 and technetium-99m, two commonly used PET and SPECT radionuclides, respectively, possess different emission profiles, offering the potential for imaging one in the presence of the other. However, the impact of the presence of each radionuclide on scanning the other could be significant and lead to confounding results. Here we use combinations of ^18^F and ^99m^Tc to explore the challenges posed by dual tracer PET/SPECT imaging, and investigate potential practical ways to overcome them.

**Methods:**

Mixed-radionuclide ^18^F/^99m^Tc phantom PET and SPECT imaging experiments were carried out to determine the crossover effects of each radionuclide on the scans using Mediso nanoScan PET/CT and SPECT/CT small animal scanners.

**Results:**

PET scan image quality and quantification were adversely affected by ^99m^Tc activities higher than 100 MBq due to a high singles rate increasing dead-time of the detectors. Below 100 MBq ^99m^Tc, PET scanner quantification accuracy was preserved. SPECT scan image quality and quantification were adversely affected by the presence of ^18^F due to Compton scattering of 511 keV photons leading to over-estimation of ^99m^Tc activity and increased noise. However, ^99m^Tc:^18^F activity ratios of > 70:1 were found to mitigate this effect completely on the SPECT. A method for correcting for Compton scatter was also explored.

**Conclusion:**

Suitable combinations of injection sequence and imaging sequence can be devised to meet specific experimental multi-tracer imaging needs, with only minor or insignificant effects of each radionuclide on the scan of the other.

## Introduction

Individually, PET and SPECT tracers allow us to probe the underlying molecular characteristics of physiological processes, one mechanism at a time. The ability to image one tracer in the presence of another—dual radionuclide PET/SPECT imaging—enables different modality tracers to be present simultaneously, thus allowing multiple processes to be imaged in the same subject, within a much shorter period of time (removing the need to wait for tracer decay). For example, radionuclides fluorine-18 (PET) and technetium-99m (SPECT) each possess different emission profiles and, in theory, can be imaged in the presence of the other, but the impact of each on scanning of the other (i.e. the SPECT and PET scans respectively), could be significant and lead to confounding results.

Acquiring a PET image in the presence of a SPECT radionuclide may introduce additional dead-time (the time after each photon is detected by the scanner during which the system is not able to record another event). Photons emitted from decaying ^99m^Tc nuclei are not coincident, and their energy of 140 keV is much lower than the 511 keV PET scanner energy window, so do not contribute to the image data acquired. However, they do interact with the PET detectors, and at high enough flux, can potentially prevent true coincidence events from the positron emitter being recorded. One study in mice showed PET signal loss of 12% due to increased dead-time when ^99m^Tc was present in an almost 10-fold higher activity compared to ^18^F [1]. It is worth noting that this phenomenon is not specific to mixed radionuclide effects, and dead-time is generally recognized as a performance-limiting factor at high concentrations of PET tracers [2].

Performing a SPECT scan in the presence of a PET radionuclide can also be problematic. If ^18^F is present during a ^99m^Tc SPECT scan, a proportion of the photons from ^18^F positron annihilation will enter the SPECT ^99m^Tc energy window (140.5 keV, ±10% i.e. 20% width) due to Compton scattering— the scattering of a photon by a charged particle, resulting in a decrease in energy and change in trajectory of the photon. Previous studies have shown that this down-scatter can generate significant noise and artifacts in the SPECT image [1]. A clinical study showed that the simultaneous use of ^99m^Tc-sestamibi and [^18^F]FDG (with a ^99m^Tc:^18^F ratio of 3.2:1) resulted in a <6 % increase in the apparent ^99m^Tc count rate due to down-scatter from ^18^F [3]. This overestimation can be corrected for on clinical scanners by the use of auxiliary energy windows; such methods use the signal in parts of the spectrum outside the photopeak window to estimate an amount of signal to subtract from the imaging window to correct for scatter [4, 5]. However, these methods may be difficult to implement in a preclinical setting due to the low volume of the scatter medium (mice, rats etc.) and hardware and software constraints.

Here, we explore some of the challenges posed by dual tracer PET/SPECT preclinical imaging using radionuclides ^18^F and ^99m^Tc as examples, and investigate potential practical ways to overcome these obstacles by appropriate experimental design. Mixed-radionuclide ^18^F/^99m^Tc phantom experiments were carried out to determine the crossover effects of each radionuclide on the scans, and ultimately, to help design the optimal protocol for *in vivo* dual radionuclide preclinical imaging using ^18^F and ^99m^Tc.

## Methods

### PET Scanner Phantoms

Plastic syringes (5mL) were filled with either ^18^F only (5 MBq), ^99m^Tc only (5 MBq), or mixtures of ^18^F (5 MBq) and increasing amounts of ^99m^Tc (5, 50, 100, 150, 200, 250, 350 MBq). Activities of 5 MBq ^18^F and < 200 MBq ^99m^Tc reflect routine protocols avoiding count-rate limitations of each modality separately. Volumes were made up to 3 mL by addition of water. Radioactivity in each syringe was measured using a dose calibrator (Capintec, Ramsey, NJ, USA), calibrated to the national standard. First the syringe was filled with ^18^F only and the activity measured. Then the required activity of ^99m^Tc was prepared in a microcentrifuge tube, measured in the dose calibrator, and transferred to the syringe. Residual ^99m^Tc activity in the needle and microcentrifuge tube was subtracted from the measured activity. Syringes were inverted several times for uniform distribution of radioactivity. Each syringe was placed in the pre-calibrated nanoPET/CT (Mediso, Budapest, Hungary; system sensitivity 4.67% for a 350-650 keV energy window and 4 ns coincidence window [2]). A 15 min PET scan was acquired with a 5 ns coincidence window in 1-5 coincidence mode. Subsequently, a CT scan was obtained for attenuation correction with a 55 kVp X-ray source, 600ms exposure time in 180 projections over ~6 min. PET images were reconstructed in Nucline v.0.21 using Tera-Tomo 3D reconstruction with 4 iterations, 6 subsets, 1-3 coincidence mode, voxel sized 0.4 mm (isotropic), energy window 400-600 keV with attenuation, and scatter correction. Images were analyzed in VivoQuant™ v.3.5, patch 2 software (Invicro LLC., Boston, USA). The activity was determined within a cylindrical ROI slightly larger than the syringe. The same cylindrical ROI was used for each scan and translated or rotated to accommodate variations in the placement of each syringe. The resulting activity from the PET/CT scan was compared to the decay-corrected activity measured in the dose calibrator. Quantitative assessment of image quality was assessed by calculating the coefficient of variation for each image: a small spherical ROI was drawn over the images and the standard deviation within the ROI was divided by the mean within that ROI (Supplementary Figure 1).

### SPECT Scanner Phantoms

Plastic microcentrifuge tubes (1.5mL) were filled with either ^99m^Tc only (1 MBq), ^18^F only (1 MBq), or mixtures of ^99m^Tc (1, 10, 30, 50, 70 MBq) and ^18^F (1 MBq) to achieve ^99m^Tc:^18^F ratios ranging from 1:1 to 70:1. Volumes were made up to 1mL by addition of water. Radioactivity in each tube was measured using a dose calibrator (Capintec, Ramsey, NJ, USA), calibrated to the national standard. First the tube was filled with ^18^F only and the activity measured. Then the required activity of ^99m^Tc was prepared in a second microcentrifuge tube, measured in the dose calibrator, and transferred to the first tube. Residual ^99m^Tc activity in the needle and second tube was subtracted from the measured activity. Tubes were inverted several times and vortexed for uniform distribution of radioactivity. Each tube was placed in the pre-calibrated nanoSPECT/CT Silver Upgrade (Mediso Ltd., Budapest, Hungary) and imaged with acquisition time 15min, frame time of 35 s using a 4-head scanner with 4 × 9 (1mm) pinhole collimators in helical scanning mode, and CT images with a 55 kVp X-ray source, 1,000ms exposure time in 180 projections over approximately 9 min. Images were reconstructed in a 256 × 256 matrix, voxel size 0.3mm (isotropic) using HiSPECT (ScivisGmbH) a reconstruction software package, and images were fused using proprietary VivoQuant™ v.3.5, patch 2 software (Invicro LLC., Boston, USA). The resulting activity from the SPECT/CT scan was compared to the decay-corrected activity measured in the dose calibrator.

### Compton Scatter Data Correction

A method to correct for the effects of 511 keV scattered photons on SPECT image quantification and quality was explored. A set of phantom experiments was performed on the nanoSPECT/CT as proof-of-concept. To obtain an ^18^F scatter map, a microcentrifuge tube containing ^18^F only (2 MBq, 1 mL) was placed in a 50mL Falcon tube of water (to mimic preclinical scatter conditions) and a 15min SPECT scan was acquired, followed by a CT scan. Next, a tube containing a mixture of ^99m^Tc (50 MBq) and ^18^F (2 MBq) was placed in a 50mL Falcon tube on the scanner and a 15min scan was acquired, followed by a CT scan. Finally, a tube containing ^99m^Tc only (50 MBq) was placed on the scanner and a 15min scan was acquired. The scatter correction was applied by subtracting the ^18^F-only SPECT scatter map from the mixed-radionuclide SPECT scan, according to the programming code (Python 3) in Supplementary Figure 2A. Automatic scatter correction was applied by scaling the voxel values in both the ^18^F-only scan and the mixed-radionuclide scan using their “COUNTS Real World Value Slope” as determined by the SPECT scanner calibration saved in the original dicom files. This allowed a matrix subtraction to be performed where each voxel value corresponded directly with real world counts, as measured by the SPECT. The images were then converted back using the Value Slope for the original mixed-radionuclide image (Supplementary Figure 2B). Both the original and scatter-corrected images were analyzed in VivoQuant™ v.3.5, patch 2 software (Invicro LLC., Boston, USA). The activity in each image was determined using the same ROI, within a region slightly larger than the microcentrifuge tube. The activity quantified in both images was compared to the decay-corrected activity measured in the dose calibrator.

It is important to note that the radioactivity quantities used in these experiments reflect the doses appropriate for our scanner system specifications; tested doses may need to be adjusted for other systems.

## Results

### Effect of ^99m^Tc on PET Scans

The effect of the presence of SPECT radionuclide ^99m^Tc on ^18^F PET scans was assessed by the use of phantoms. The PET scanner was calibrated prior to the start of the study following manufacturer procedures; ^18^F-only phantoms measured in the dose calibrator and PET scanner showed good agreement (within 2%).

**FIGURE 1:**
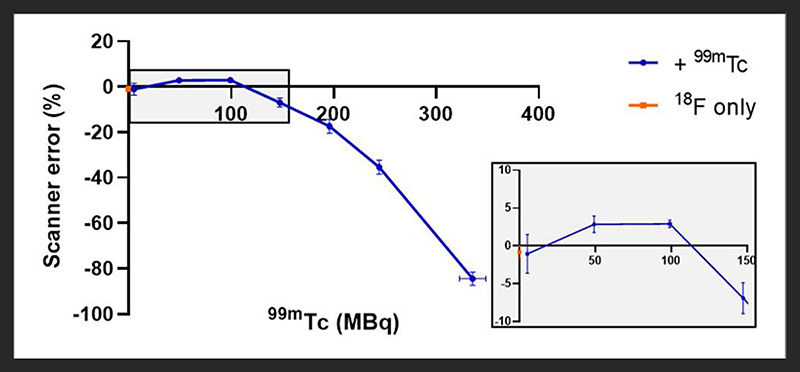
Effect of different amounts of ^99m^Tc on the accuracy of PET scanner quantification of 5 MBq ^18^F. The effect on scanner quantification was assessed by comparing the amount of ^18^F measured by the dose calibrator to that measured by the PET scanner; *n* = 3, mean ± SD. Gray box inset provides zoom of 0-150 MBq region.

**FIGURE 2:**
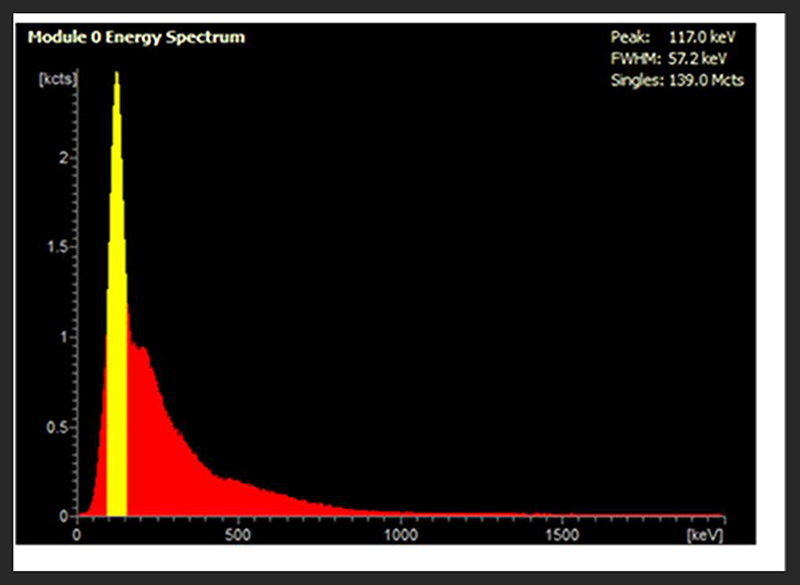
Live acquisition energy spectrum obtained during a PET scan of a mixed radionuclide phantom containing 5 MBq ^18^F and 250 MBq ^99m^Tc. The yellow peak at 140 keV corresponds to the energy of ^99m^Tc y photons, which reduces detection of ^18^F coincident photons at 511 keV

In the presence of up to 100 MBq ^99m^Tc, 5 MBq ^18^F was accurately quantified by the PET scanner: an over-estimation of < 5% activity was observed in the presence of 50 and 100 MBq ^99m^Tc (Figure 1). At higher activities of ^99m^Tc, PET scanner quantification became less accurate and consistently under-estimated the amount of ^18^F present. At 150 MBq of ^99m^Tc, ^18^F quantification was underestimated by < 10% and became progressively worse with increasing amounts of ^99m^Tc: at 350 MBq the scanner was underestimating activity of ^18^F by > 80% (Figure 1). The live acquisition energy spectrum (Figure 2, Supplementary Figure 3) showed that at these higher activities of ^99m^Tc, photons at 140 keV (attributable to ^99m^Tc decay) overwhelmed the detection of 511 keV photons originating from ^18^F decay suggesting that the dead-time correction could not cope with the increased singles rate. Note that the true counts decrease considerably when adding ^99m^Tc to ^18^F (Supplementary Tables 1, 2). However, activity quantification showed low errors up to 100 MBq added ^99m^Tc (Figure 1) due to the intrinsic dead-time correction of the scanner. Note also that both ^99m^Tc only and water have similar numbers of true counts which originate from the intrinsic radiation of the LYSO:Ce crystals.

**FIGURE 3:**
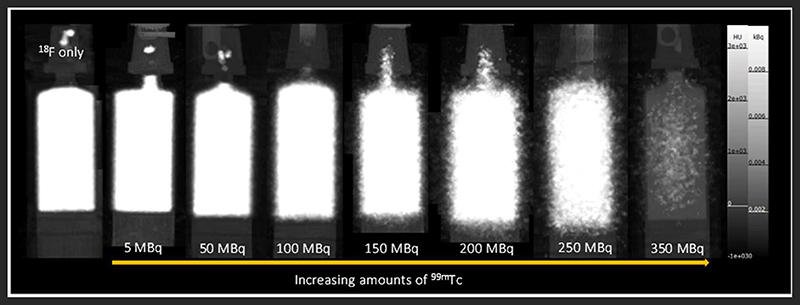
PET-CT MIPs of mixed-radionuclide ^18^F + ^99m^Tc phantoms. Each syringe contains ^18^F (5 MBq) mixed with increasing amounts of ^99m^Tc (0-350 MBq). An ^18^F only (5 MBq) control is included for comparison.

**FIGURE 4:**
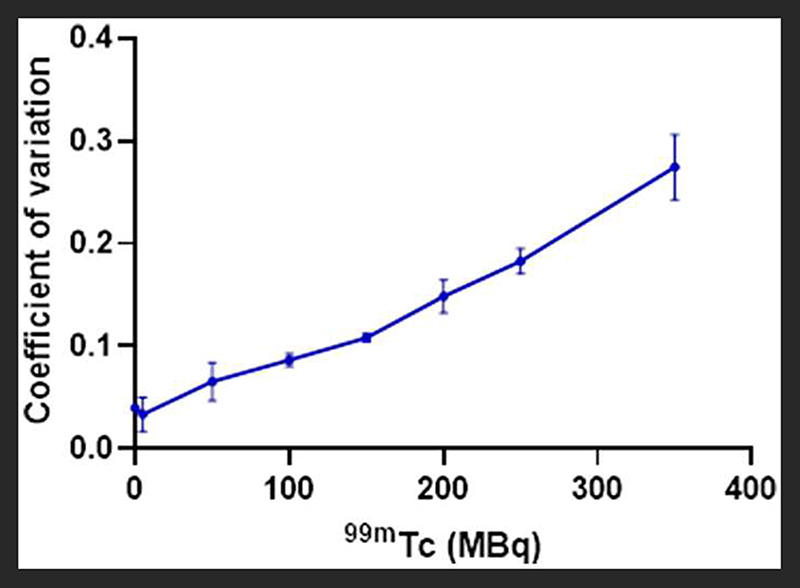
Effect of increasing amounts of ^99m^Tc on the PET image quality of 5 MBq ^18^F, quantified by calculating the coefficient of variation within an ROI for each image (SD/mean); *n* = 3.

The effect of ^99m^Tc on PET image quality was examined (qualitatively) by observing image noise present with increasing ^99m^Tc activity in the field-of-view. PET image quality was maintained in the presence of 5 MBq ^99m^Tc when compared to its ^18^F-only control (Figure 3). Addition of 50 MBq ^99m^Tc caused image quality (in terms of signal-to-noise) to deteriorate noticeably, with images becoming more diffuse and lacking in definition. Image quality became progressively worse with increasing amounts of ^99m^Tc (Figure 3). These qualitative observations were supported by quantitative analysis of the images: the coefficient of variation for each image increased with increasing amounts of ^99m^Tc present (Figure 4).

### Effect of ^18^F on SPECT Scans

The effect of the presence of PET radionuclide ^18^F on ^99m^Tc SPECT scans was assessed by the use of phantoms. The SPECT scanner calibration was checked prior to the start of the study: ^99m^Tc-only phantoms measured in the dose calibrator and SPECT scanner showed good agreement (within 5%).

**FIGURE 5:**
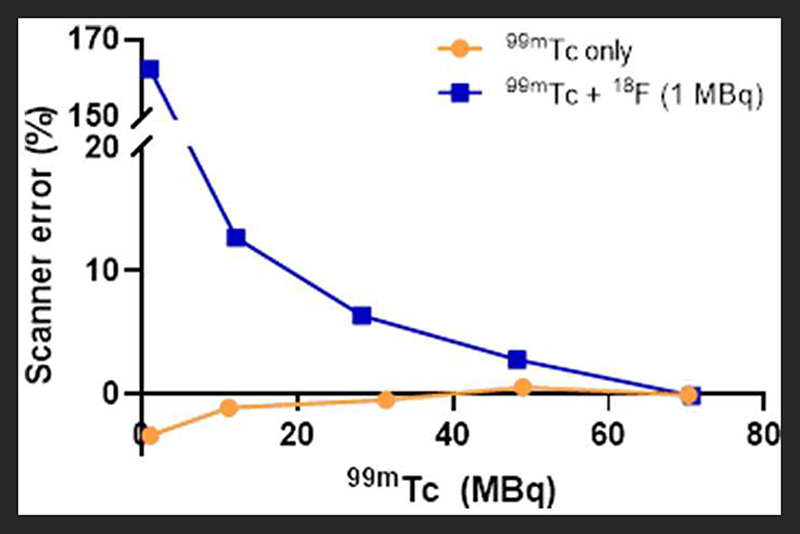
Effect of ^18^F (1 MBq) on the accuracy of SPECT scanner quantification of increasing amounts of ^99m^Tc. The effect on scanner quantification was assessed by comparing the amount of ^99m^Tc measured by the dose calibrator to that measured by the SPECT scanner.

**FIGURE 6:**
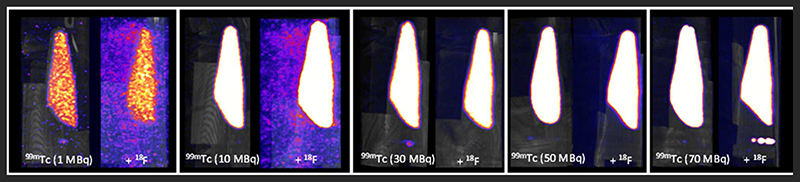
SPECT-CT MIPs of ^99m^Tc only and mixed-radionuclide ^99m^Tc + ^18^F phantoms. Each tube contained either ^99m^Tc only (1, 10, 30, 50, 70 MBq) or ^99m^Tc (1, 10, 30, 50, 70 MBq) mixed with ^18^F (1 MBq). All images scaled to the same threshold and intensity.

**FIGURE 7:**
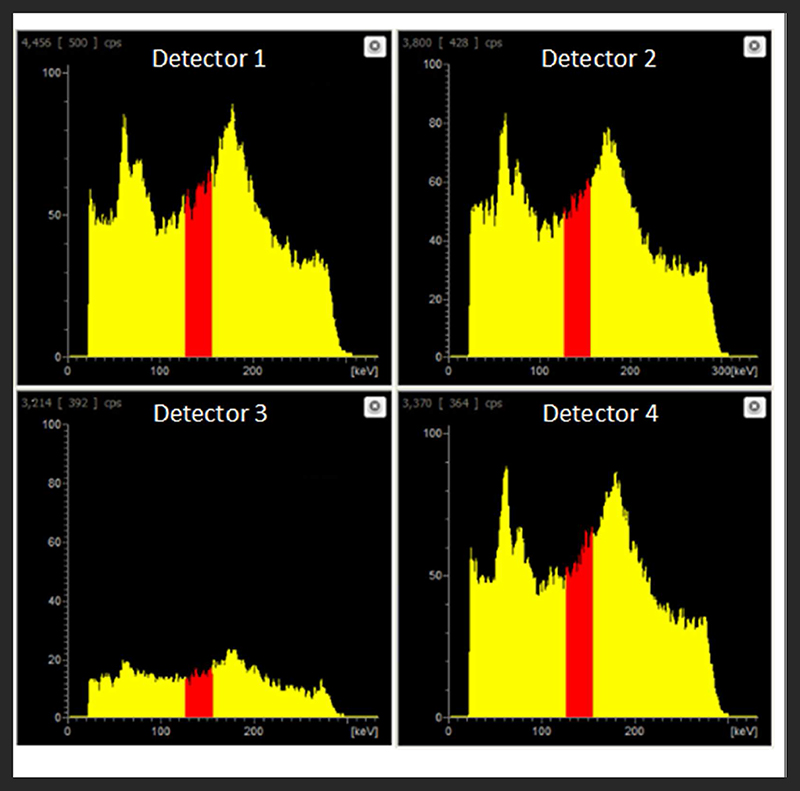
Live acquisition energy spectrum obtained during a SPECT scan of an ^18^F-only phantom (1 MBq). A range of energies is evident, including energies in the 140.5 keV ±10% (i.e. 20% width) ^99m^Tc energy window (red).

At equivalent activities of ^99m^Tc and ^18^F (1:1), quantification of ^99m^Tc was poor, with the scanner overestimating ^99m^Tc activity by > 150% (1 MBq vs. 2.75 MBq) (Figure 5). A 10-fold increase in the activity of ^99m^Tc relative to ^18^F dramatically improved scanner quantification accuracy, reducing ^99m^Tc activity overestimation to 10% (Figure 5). Further increases in the quantity of ^99m^Tc incrementally improved scanner quantification accuracy in the presence of 1MBq^18^ F. The adverse effects of ^18^F on scanner quantification accuracy were mitigated completely when ^99m^Tc was in 70-fold excess compared to ^18^F (Figure 5). SPECT image quality was also affected by the presence of ^18^F, with high levels of noise observed at ^99m^Tc:^18^F ratios of 1:1 and 10:1 (Figure 6). At ratios of 30:1 and above, noise levels observed qualitatively in the images were significantly reduced and images became sharper (Figure 6). The acquisition energy spectrum of an ^18^F-only phantom on the SPECT scanner showed detection of a range of photon energies, including some in the 140 ± 10% (20% width) keV ^99m^Tc energy window (Figure 7).

### Compton Scatter Data Correction

**FIGURE 8:**
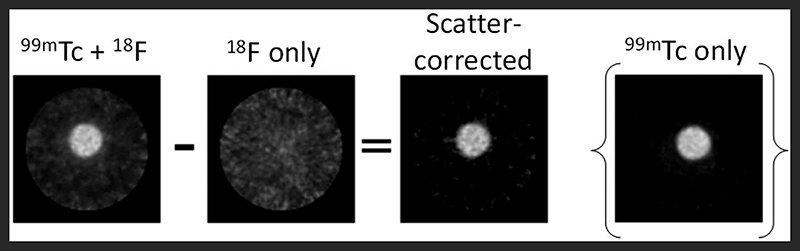
Proof-of-concept for SPECT scatter data correction method. The ^18^F-only scatter map image was subtracted from the ^99m^Tc + ^18^F mixed-radionuclide image to achieve the final scatter-corrected image. The ^99m^Tc-only image is included for comparison.

The SPECT scanner calibration was checked prior to the start of the study: ^99m^Tc-only phantoms measured in the dose calibrator and SPECT scanner showed good agreement (within 1%): a ^99m^Tc-only sample measured 50.8 MBq and 51.0 MBq in the dose calibrator and the SPECT scanner, respectively. The mixed-radionuclide sample containing ^18^F (2 MBq) plus ^99m^Tc (50.7 MBq, measured by dose calibrator) was quantified as 55.84 MBq on the SPECT scanner in the 140 ± 10% keV window, an over-estimate of 10% resulting from the contribution of Compton-scattered 511 keV photons to the 140 keV window. Upon subtraction of the ^18^F-only phantom counts from the mixed ^18^F+^99m^Tc counts, the resulting “scatter-corrected” data were quantified at 51.89 MBq, an over-estimation of only 2%. The coefficient of variation for the original image and scatter-corrected image was 0.098 and 0.102, respectively. The resulting image was visibly similar to the ^99m^Tc-only control image, showing a substantial reduction in noise and comparable activity compared to the mixed-radionuclide image (Figure 8). This could be implemented in practice as data correction: initially a SPECT scan of the ^18^F present is acquired before injection of ^99m^Tc, to establish the ^18^F down-scatter contribution; following injection of ^99m^Tc and acquisition of the SPECT scan, the ^18^F down-scatter component is subtracted to provide an accurate ^99m^Tc uptake distribution.

## Discussion

Our specific motivation for this work originated from the need to compare directly two PET tracers for the same biological target, both labeled with ^18^F, to understand subtle differences in behavior between the two tracers *in vivo*. Since the two tracers were labeled with the same PET radionuclide, and hence had identical physical emission profiles, they could not be compared *simultaneously* in the same animal. A consecutive imaging protocol (administration and PET imaging of the first tracer, followed by administration and PET imaging of the second tracer in the same animal) was possible, in theory. However, the need to allow the first tracer to decay sufficiently to prevent residual activity interfering with the second scan, combined with limits on animal exposure to anesthesia and recovery time (our specific animal license requires a minimum of 3 h between mouse recovery and being re-anesthetized) means the imposed delay between tracer administration could lead to significant physiological changes (effects of anesthesia, metabolism, tumor size etc.) in the animal between the two scans. Similarly, evaluating each tracer in a different animal introduces inherent variability between mice, thus no longer maintaining a controlled environment for accurate tracer comparison.

Our solution was to adopt a paired-control approach, using a single SPECT (^99m^Tc) tracer (for the same biological target) in conjunction with each PET tracer, where each tracer can be imaged in the presence of the other because ^18^F and ^99m^Tc possess different emission profiles. Thus, the need to understand the impact of each radionuclide on the scanning of the other presented itself, and mixed-radionuclide ^18^F/^99m^Tc phantom experiments were carried out to determine the crossover effects of each radionuclide on the scans. Of course, dual radionuclide PET/SPECT imaging is not only relevant to our niche example; it is of value more generally, to enable evaluation of different, but related, biological systems at (almost) the same time using different tracers labeled with different radionuclides.

Firstly we examined the effect of ^99m^Tc on ^18^F PET scans. Dead-time effects are observed on the PET scanner when there is too much of any radionuclide. A previous Noise Equivalent Counting (NEC) study performed on our PET scanner showed count-rate peaks at 430 kcps at 36 MBq and 130 kcps at 27 MBq for ^18^F in mouse and rat phantoms, respectively [2]. The injected activity for a mouse in our PET scanner is typically 2-10 MBq; 5 MBq ^18^F was used in this phantom experiment, which is well below the NEC peaks, and therefore quantification is not affected by dead-time when this quantity of ^18^F alone is used. However, when combined with ^99m^Tc, we must assess the contribution of ^99m^Tc photons emitted to scanner dead-time, and hence the effects on PET image quality and quantification. We see that when combined with lower amounts of ^99m^Tc (<100 MBq), ^18^F quantification is unaffected, but 140 keV photon contribution from higher amounts of ^99m^Tc (>150 MBq) prevents coincident 511 keV PET photons being recorded, causing the scanner to significantly underestimate ^18^F activity. In practice, a typical ^99m^Tc SPECT scan requires only 10-40 MBq injection. These results enable us to assess the feasibility of a hypothetical imaging protocol as follows: (i) administration of SPECT tracer (^99m^Tc, 40 MBq) (ii) SPECT image acquisition (1 h) (iii) administration of PET tracer (^18^F, 5 MBq) (iv) PET image acquisition (1 h), in the presence of ^99m^Tc. Our phantom studies show that at these levels of activity, ^18^F PET quantification is not affected by the presence of ^99m^Tc. However, even at levels of ^99m^Tc where quantification accuracy is maintained, PET image quality does appear to be compromised. In the presence of 50 MBq ^99m^Tc, images becomes a little more diffuse with reduced definition. While this slight decrease in image quality is unlikely to be problematic for the majority of imaging scenarios, it could make identification and visualization of very small biological structures (e.g. tumor metastases) more difficult.

Next we examined the effect of ^18^F on ^99m^Tc SPECT scans. In the presence of ^18^F, ^99m^Tc SPECT image quantification and quality were affected by Compton scatter, leading to an overestimation of ^99m^Tc and increase in noise. However, when ^99m^Tc activity was 70-fold higher than ^18^F this over-estimation could be mitigated completely and image quality preserved.

In practice, a good 1 h dynamic PET scan can be achieved with 3 MBq ^18^F alone and, taking into account decay (t_1_/_2_ = 109.8min), no more than 2 MBq of ^18^F tracer would be residual in the animal after the 1 h scan. Our results enable assessment of the feasibility of an alternative hypothetical imaging protocol, as follows: (i) administration of PET tracer (^18^F, 3 MBq) (ii) PET image acquisition (1 h) (iii) administration of SPECT tracer (^99m^Tc, > 140 MBq, to maintain 70:1 ^99m^Tc:^18^F ratio) (iv) SPECT image acquisition (1 h) in the presence of ^18^F. Our phantom studies show that at these radionuclide activity ratios, ^99m^Tc SPECT image quantification and quality is not affected by the presence of ^18^F. However, although we can control this radionuclide activity ratio at the time of SPECT tracer injection, we cannot know if this 70:1 ratio is maintained *in vivo*—the biodistribution of both PET and SPECT tracers would need to be similar for this to be true. This, plus the need for otherwise unnecessarily high levels of radioactivity, makes this protocol an inferior option.

From a biological perspective, it would be preferable to co-administer both tracers at the same time, but based on our findings, there is no combination of imaging sequence and tracer quantity (without waiting several hours for radionuclide decay) that would allow the two radionuclides to be injected *simultaneously* and each scanned in the presence of the other, without having a major effect on image quality and quantification. Furthermore, images of each tracer at the same time after injection could not be obtained with simultaneous injection.

We also explored simple computational methods for correcting for the effects of Compton scatter on the SPECT scanner. One approach involves using the ^18^F-only PET scan to identify sources of ^18^F, and then subtracting this from the mixed-radionuclide SPECT scan. This process becomes quite complex because we are attempting to combine data collected from two different and essentially unrelated pieces of equipment; differences between the PET and SPECT scanners must be accounted for and this requires additional calibration steps to be done for each set of measurements obtained on the scanners. An additional complication of this method is the need to maintain animal position between the two scanners so that images can be accurately co-registered. Even with the most careful transfer between scanners (the animal bed is compatible with both scanners and could be transferred without removing the mouse), a registration step would be required using the CT scans of the mice to obtain a “best case” match for the scatter map. While it would be possible to produce a warp field for the mouse shift in position, this quickly becomes an overly-complex solution. A more practical approach might therefore be achieved using the SPECT scanner directly to obtain an ^18^F scatter map, prior to injection of ^99m^Tc. This method would involve incorporating an additional SPECT scan into the scanning protocol and makes the assumption that scatter present in the ^18^F-only scatter map is comparable to that obtained when the SPECT tracer is also present (i.e. no change in biodistribution between SPECT scans). The scatter map must also be corrected for radionuclide decay between scans. Our proof-of-concept phantom experiment demonstrated that this scatter-correction method could be used easily and successfully to remove noise caused by Compton scattering of ^18^F photons. The proposed preclinical imaging protocol would then be as follows: (i) administration of PET tracer (^18^F) (ii) PET image acquisition (iii) SPECT image acquisition (to obtain ^18^F scatter map) (iv) administration of SPECT tracer (^99m^Tc) (v) SPECT image acquisition (in the presence of ^18^F) (vi) scatter map image subtraction. This approach builds the scatter map acquisition into the scanning protocol, and would therefore be applicable to any PET/SPECT radionuclide combination, any radionuclide activity, and any scanner model, without requiring calibration for each experiment, making this method an attractive option for dual radionuclide PET/SPECT imaging.

The ability to obtain accurate parallel scans with PET and SPECT tracers would allow the limits of molecular imaging to be extended and would be useful for comparison of different radiotracers or to image multiple related molecular processes simultaneously to obtain a deeper understanding of interlinked processes. Examples could include temporospatial mapping of anti-cancer drug delivery (e.g. via liposomal formulation) and response in relation to the disease site [6], and in advanced cell-based therapies where the trafficking of cells to disease sites has to be quantified alongside mapping of the disease and response to therapy. Similarly, SPECT imaging of the biodistribution of therapeutic radionuclides that emit both imageable gamma photons (e.g. ^177^Lu, ^67^Cu, ^188^Re) could be performed alongside PET imaging (e.g. with [^18^F]FDG or other tracer) of metabolic response to therapy. It would also enable imaging of multiple molecular characteristics of disease in an animal to obtain metabolic and gene expression profiles or characterize diseases such as cancer where heterogeneity is to be expected.

## Conclusion

Sequential PET and SPECT data acquisition whilst both positron- and gamma-emitters are present is feasible under certain conditions without substantial influence on image quantification accuracy. Suitable combinations of injection sequence and imaging sequence can be devised to match the biological requirements of a particular experiment, using existing commercial small animal PET and SPECT scanners, with only minor or insignificant effects of each radionuclide on the scan of the other. However, simultaneous tracer injection is not feasible under any combination of imaging sequence and tracer quantity, and does not allow images of each tracer to be obtained at the same time after injection.

## Supplementary Material

Supplementary material

## Data Availability

The datasets generated for this study are available on request to the corresponding author.
