# Supplementary material for: Protocols for Dual Tracer PET/SPECT Preclinical Imaging
Source: Front Phys. Author manuscript; Available in PMC 2021 Jun 9. (PMC7610950; doi:10.3389/fphy.2020.00126)
Supplement: Supplementary material [file EMS126868-supplement-Supplementary_material.DOCX]

Supplementary Material


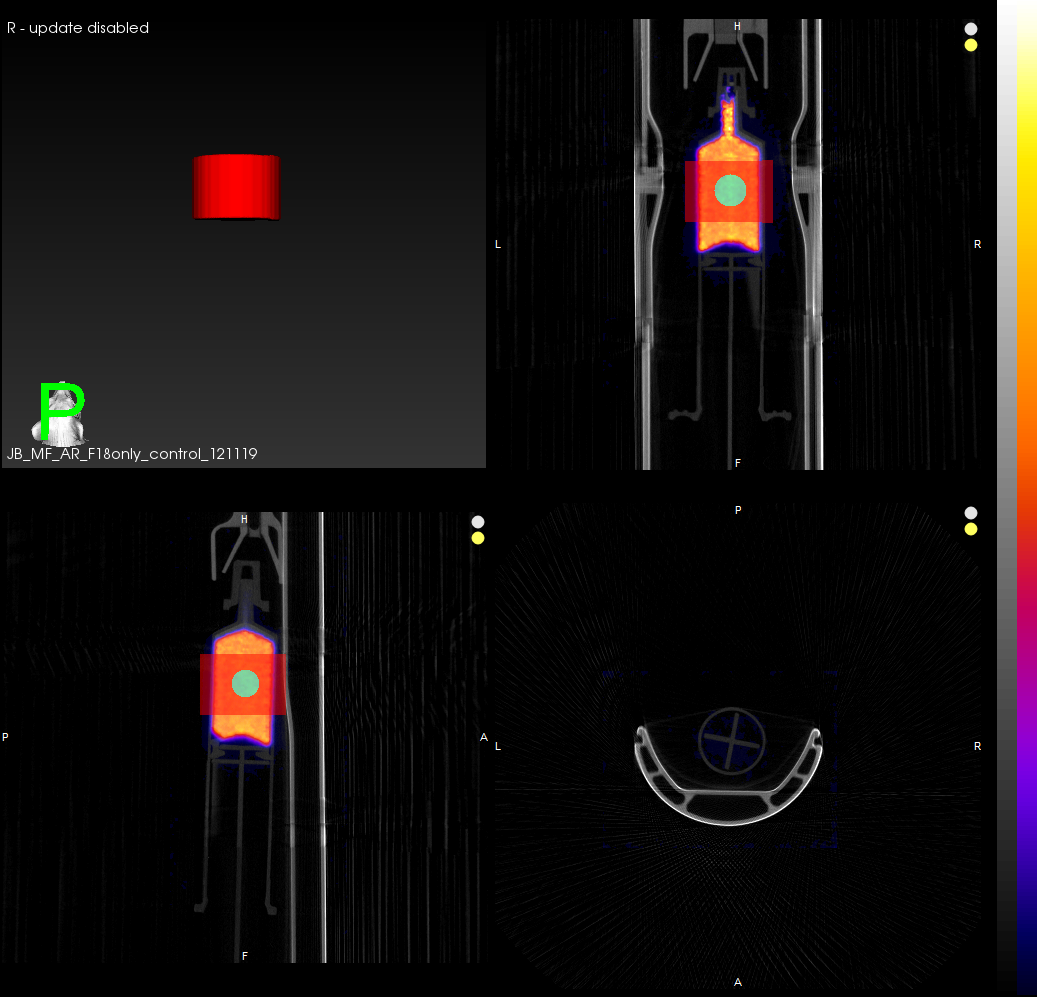


**Supplementary Figure 1.** ROI template used to calculate the coefficient of variation (SD/mean) as a method for quantifying PET image quality.

Code sequence

import sys, os

import numpy as np

import SimpleITK as sitk

def subtract(path_1, path_2):

# lets assume every folder in this folder has one "SPECT" folder with one file

for f in os.listdir(path_1): scan_1 = sitk.ReadImage(path_1+f); break

for f in os.listdir(path_2): name = f; scan_2 = sitk.ReadImage(path_2+f); break

scan_array_1 = sitk.GetArrayFromImage(scan_1)

scan_array_2 = sitk.GetArrayFromImage(scan_2)

print np.zeros([2,128,128]).shape

scan_array_1 = np.append(scan_array_1, np.zeros([2,128,128]), axis=0)

print scan_array_1.shape

result_array = scan_array_1 - scan_array_2

result_image = sitk.GetImageFromArray(result_array.clip(min=0))

result_image.CopyInformation(scan_2)

sitk.WriteImage(sitk.GetImageFromArray(scan_array_1), '1.nii')

sitk.WriteImage(sitk.GetImageFromArray(scan_array_2), '2.nii')

sitk.WriteImage(result_image, 'out.nii')

return

def main():

if len(sys.argv) > 2:

subtract(sys.argv[1], sys.argv[2])

else:

print("provide path to import files")

return

if __name__== "__main__":

main()

import sys, os

import numpy as np

import SimpleITK as sitk

"""

Imports a folder of scans and prints total counts

Usage "python process.py [FOLDER OF SCANS]"

"""

def files_to_total_counts(path):

    # lets assume every folder in this folder has one "SPECT" folder with one file

    for f in os.listdir(path):

        folder = path+"/"+f+"/SPECT"

        scan = os.listdir(folder)[0]

        scan = sitk.ReadImage(folder + "/" + scan)

        scan_array = sitk.GetArrayFromImage(scan)

        print f

        print np.sum(scan_array)

    return

def main():

    if len(sys.argv) > 1:

        files_to_total_counts(sys.argv[1])

    else:

        print("provide path to import files")

        return

if __name__== "__main__":

    main()

**Supplementary Figure 2a**. Code sequence for SPECT scanner scatter correction subtraction, performed in Python 3.

Real World Value Slope = 3218823000

COUNTS

(0040,9225) FD 0.0048710391595989918

3218823000 * 0.0048710391595989918 = **15679.012k counts**

MBq

(0040,9225) FD 0.016122596655156089

3218823000 * 0.016122596655156089 = 51895784Bq = **51.89 MBq**

**Supplementary Figure 2b**. Calculation for converting image voxel values to MBq and Counts using “Real World Value Slope”: after subtraction the resulting corrected image was saved in NIfTI-1 data format (.nii) for analysis. The voxel value sums in the region of interest were converted manually to MBq and Counts using the "Real World Value Slope" from the pre-correction dicom using the above calculation. This was done as the software used to draw the region of interest did not do this automatically using NifTI-1 files.

**Supplementary Figure 3.** Live acquisition energy spectra obtained during PET scans of radioactive phantoms containing 5 MBq ^18^F or mixed-isotope phantoms containing 5 MBq ^18^F and increasing amounts of ^99m^Tc. The 511 keV peak becomes increasingly overwhelmed by the 140 keV peak as the amount of ^99m^Tc present increases.

| **Activity at start of scan** | **True counts (Mcts)** | **Random counts (Mcts)** |
| --- | --- | --- |
| ^18^F 5 MBq only | 120 | 1 |
| ^18^F 5 MBq + ^99m^Tc 5 MBq | 110 | 1 |
| ^18^F 5 MBq + ^99m^Tc 50 MBq | 52 | 0.7 |
| ^18^F 5 MBq + ^99m^Tc 100 MBq | 25 | 0.7 |
| ^18^F 5 MBq + ^99m^Tc 150 MBq | 13 | 0.9 |
| ^18^F 5 MBq + ^99m^Tc 200 MBq | 7.0 | 1.1 |
| ^18^F 5 MBq + ^99m^Tc 250 MBq | 3.7 | 1.5 |
| ^18^F 5 MBq + ^99m^Tc 350 MBq | 1.3 | 2 |
| ^99m^Tc 5 MBq only | 0.030 | 0.002 |
| water | 0.035 | 0.002 |

**Supplementary Table 1**. True coincident counts and random counts for phantoms containing 5 MBq ^18^F and increasing amounts of ^99m^Tc, or 5 MBq ^99m^Tc only or water only. Values are approximated for easy visualisation, accurate values are reported in Supplementary Table 2.

Note that the true counts decrease considerably when adding ^99m^Tc to ^18^F. However, activity quantification showed low errors up to 100 MBq added ^99m^Tc (Figure 1) due to the intrinsic dead-time correction of the scanner. Note also that both ^99m^Tc only and water have similar numbers of true counts which originate from the intrinsic radiation of the LYSO:Ce crystals (1).

| **^18^F activity**  **(MBq)** | **^99m^Tc activity (MBq)** | **True counts** | **Random counts** |
| --- | --- | --- | --- |
| 3.62 | 0 | 83,200,482 | 670,337 |
| 4.93 | 0 | 121,939,453 | 1,129,478 |
| 4.77 | 0 | 118,015,750 | 1,058,272 |
| 4.68 | 5.24 | 105,616,859 | 956,392 |
| 4.85 | 4.25 | 111,517,383 | 1,029,977 |
| 5.96 | 6.02 | 129,016,005 | 1,437,230 |
| 4.79 | 49.44 | 50,192,062 | 661,024 |
| 4.88 | 49.43 | 52,735,821 | 666,626 |
| 5.03 | 48.65 | 58,008,137 | 796,782 |
| 5.03 | 97.96 | 25,877,939 | 711,208 |
| 4.79 | 98.96 | 24,207,853 | 689,377 |
| 4.78 | 100.44 | 27,754,236 | 740,646 |
| 4.85 | 147.00 | 13,252,160 | 878,290 |
| 4.70 | 148.13 | 12,955,062 | 875,668 |
| 5.19 | 147.20 | 13,792,797 | 906,655 |
| 4.94 | 197.64 | 7,031,874 | 1,162,997 |
| 5.03 | 194.92 | 7,105,759 | 1,152,842 |
| 4.60 | 194.87 | 7,361,227 | 1,135,116 |
| 4.59 | 244.69 | 3,498,039 | 1,448,124 |
| 5.00 | 244.19 | 3,986,985 | 1,518,226 |
| 4.75 | 244.77 | 4,315,315 | 1,426,862 |
| 3.96 | 356.17 | 1,442,139 | 1,962,536 |
| 4.57 | 344.31 | 1,269,843 | 2,079,024 |
| 4.56 | 344.80 | 1,522,984 | 2,107,693 |
| 0 | 5.24 | 30,264 | 1,755 |
| 0 | 0 | 35,377 | 1,520 |

**Supplementary Table 2**. ^18^F and ^99m^Tc activity (as measured by dose calibrator) decay-corrected to the start of each scan, with true and random counts for a 15 minute PET acquisition.

1. Goertzen AL, Suk JY, Thompson CJ. Imaging of weak-source distributions in LSO-based small-animal PET scanners. J Nucl Med. 2007;48(10):1692-8.
